# Low Dose Perioperative Intravenous Tranexamic Acid in Patients Undergoing Total Knee Arthroplasty: A Double-Blind Randomized Placebo Controlled Clinical Trial

**DOI:** 10.1155/2015/948304

**Published:** 2015-12-06

**Authors:** Mahdi Motififard, Mohammad Ali Tahririan, Mehdi Saneie, Sajad Badiei, Amin Nemati

**Affiliations:** ^1^Orthopedic Department, Kashani Teaching Hospital, Isfahan University of Medical Sciences, Isfahan, Iran; ^2^Students Research Center, Isfahan University of Medical Sciences, Isfahan, Iran

## Abstract

*Background and Objectives.* The null hypothesis of this study was that TA has no effect on postsurgical bleeding in patients undergoing TKA.* Methods.* This study was a double-blind randomized trial. In the first group (T) patients received 500 mg of intravenous Tranexamic acid (TA) twice (once preoperatively and once 3 hours postoperatively) and in the second group (P) they received slow infusion of normal saline as placebo. The primary outcome of the study was the level of Hb 48 hours after surgery.* Results.* Hb levels 48 hours after surgery as the primary outcome were 10.92 ± 0.97 and 10.23 ± 0.98 (g/dL) in groups T and P, respectively, and the difference was statistically significant (*P* = 0.001). Statistically significant differences were also observed in Hb levels 6 and 24 hours after surgery, the drain output 48 hours after surgery, and the number of units of packed cells transfused between study groups (*P* < 0.05). There was no significant difference in duration of hospitalization between the study groups (*P* = n.s.).* Conclusions.* The low dose perioperative intravenous TA significantly reduces blood loss, requirement for blood transfusion, and drain output in patients undergoing TKA. However, duration of hospitalization did not change significantly.

## 1. Introduction

Nowadays, total knee arthroplasty (TKA) is one of the most common procedures in orthopedic surgery and about half of the patients need blood transfusion [1, 2]. Allogeneic blood transfusion is the standard approach to increase hemoglobin (Hb) level. However, it can cause some complications such as hemolytic and nonhemolytic transfusion reactions and transmission of infectious diseases and can also increase morbidity and mortality [3, 4]. Thus, reducing the need for blood transfusion becomes a major concern for orthopedic surgeons. Several strategies have been suggested to reduce intraoperative and perioperative blood loss and as a result reduce blood transfusion [5–8]. One of them is the use of pharmacologic agents such as Tranexamic acid (TA). Pneumatic tourniquets are usually used to reduce intraoperative bleeding during TKA surgery. However, this will result in more bleeding after surgery [9]. Previous studies showed that there is a transitional activation of the fibrinolytic system after surgery which can be indices by the use of tourniquets [10]. Hence, antifibrinolytic agents could theoretically be effective in reducing bleeding.

TA is a synthetic derivative of Lysin that competitively blocks the lysine-binding sites in the plasmin and plasminogen activator molecules and its usage has become more popular in recent years [11]. Several application methods such as oral, local, and intravenous methods have been described and each one has its own advantages and disadvantages. Intravenous administration is a conventional method. There are previous studies that have focused on the administration of intravenous TA with different protocols. However, in all of these studies there were deficiencies such as lack of outcome measure, study design, or sample size. There is no consensus on its use protocol and the question still remains about the dosage, timing, and the administration method. Therefore, this study was designed. The null hypothesis of this study was that TA does not have an impaction on blood loss after TKA. This hypothesis was evaluated by measuring the decrease in Hb, the drain output volume, and the volume of blood needed to be transfused.

## 2. Materials and Methods

### 2.1. Participants and Setting

This study was a double-blind randomized clinical trial which was performed at Kashani teaching hospital, a tertiary referral center in Isfahan, Iran. During the period of study (January 2012 to March 2013) a total number of 95 patients were enrolled in the study. Eligible patients were individuals with osteoarthritis who were indicated for primary TKA. Patients with previous history of cerebrovascular disease, thromboembolism, myocardial infarction, and those who were candidates for bilateral TKA were excluded.

### 2.2. Surgical Technique and Intervention

All surgeries were performed under spinal anesthesia which was done by the anesthesiologist colleague. Two grams of intravenous Cephazolin (EXIR, Borujerd, Iran) was administered thirty minutes before the surgery.

All operations were performed in the morning between 8 am and 12 pm by a single skilled surgeon (MM) using posterior cruciate substituting cemented total knee prosthetics (Zimmer, Warsaw, IN). Surgeries were performed through an anterior skin incision from 8 centimeters above the patella to 2 centimeters distal to the tibial tubercle. A pneumatic tourniquet was used which inflated before the skin incision and deflated just before the soft tissue repair at the end of surgery. During the surgery sterilized gauzes were used for blood absorption while electrical coater and suction were established to control bleeding. At the end of surgery during soft tissue repair in extension position a low vacuum intra-articular drain was used for postoperative wound drainage which was removed 48 hours after surgery.

Eligible patients were randomly assigned to one of two groups: in the first group (Group T) they received intravenous (IV) Tranexamic acid (Caspian Tamin pharmaceutical Co., Rasht, Iran) 500 mg diluted in 100 mL of 0.9% saline chloride twice; the first dose was infused in over 10 minutes about 30 minutes before inflation of tourniquet and the second dose after staying in the recovery room for three hours. In the second group (Group P), patients received IV slow infusion of 100 mL of 0.9% sodium chloride twice. The timing was the same as that of Group T.

All patients were mobilized on the first day after surgery. DVT prophylaxis was performed by Low Molecular Weight Heparin (Clexane, Sanofi, UK) 40 mg/day postoperatively which continued for 14 days. Postoperative transfusion was done in patients with hemoglobin level less than 8 g/dL and less than 10 g/dL in patients with comorbidities and untolerated anemic symptoms. Postoperative complications including hyper coagulable states such as DVT, PTE, myocardial infarction, and cerebrovascular events were evaluated by means of clinical signs and symptoms. Further evaluations were performed when needed. Patients were discharged if they had been mobilized and the wound was dry without any discharge.

### 2.3. Measurements and Outcomes

The primary outcome of this study was the Hb level 48 hours after surgery which was evaluated by one of the study investigators (MS). The secondary outcomes were Hb levels, 6 and 24 hours after surgery, drain output during the first 48 hours after surgery, and blood product administration after surgery and duration of hospitalization. Hb levels were checked every day at 7 am. Drain output was measured during the first 48 hours after surgery. The total volume of blood drained in this period was considered as the output. Drains were replaced if filled before 48 hours.

### 2.4. Blindness and Randomization

Eligible patients were allocated to one of the two groups using “Random allocation software,” which has been used for this purpose in several previous studies [12]. This study was a double-blind one. Neither the patient nor the physician who evaluated the study outcomes knew whether a patient was in group T or P.

### 2.5. Sample Size and Statistical Analyses

Sample size was calculated using a statistical formula considering *α* = 0.05 and *β* = 0.2 expecting at least 0.5 gr/dL difference in Hb levels between the two study groups. With these inputs the sample size was calculated to be 45 in each group. Statistical analyses were performed using SPSS (SPSS, Inc., Chicago, IL, version 20). Qualitative variables were compared using the Chi-square test and presented as number (%). Quantitative variables had normal distribution and were compared using the paired *t*-test and presented as mean ± standard deviation. Written informed consent was obtained from all patients at the beginning of the study. The protocol of this study was approved in the review board of Isfahan University of Medical Sciences (number: 392114).

## 3. Results

**Figure 1:**
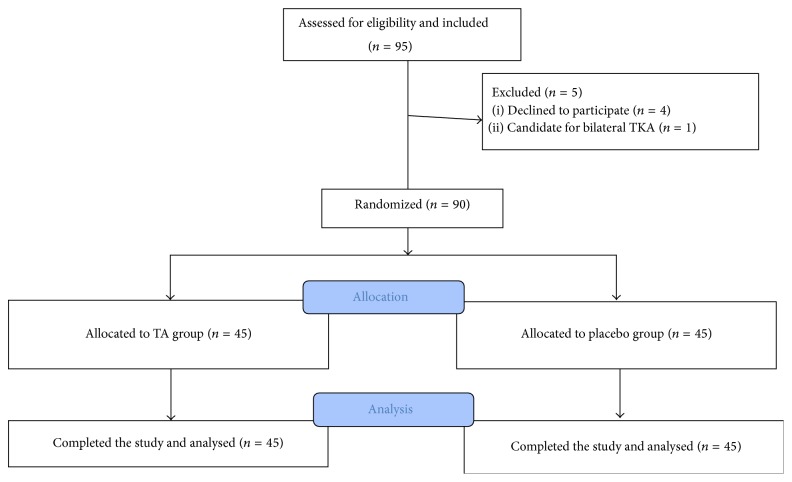
Participants' flow diagram.

Ninety-five patients were assessed for eligibility criteria. Among them four patients did not consent to participate in the research project and one patient was a candidate for bilateral TKA and, thus, the mentioned 5 patients were excluded from the study. The participant flow diagram is shown in Figure 1. There was no significant difference in the demographic variables age and sex between the study groups (Table 1).

Hb levels 48 hours after surgery were 10.92 ± 0.97 (gr/dL) and 10.23 ± 0.98 (gr/dL) for groups T and P, respectively, and the difference was statistically significant (*P* = 0.001). The differences in Hb levels between groups, 6 and 24 hours after surgery, were also found to be statistically significant (*P* = 0.04 and 0.001, resp.). Detailed data are shown in Table 2.

Drain outputs were 268.66 ± 116.68 milliliters and 478.11 ± 254.19 milliliters for groups T and P, respectively, and the difference was statistically significant (*P* = 0.001). In other words, the mean drain output of patients in group T was approximately 43% less than that of patients in group P. Patients in group P received 1.28 ± 0.75 units of packed cells while patients in group T received 0.26 ± 0.49 units (*P* = 0.001) which shows that blood transfusion in group T occurred nearly 79% less than in group P. Duration of hospitalization was 6.02 ± 2.97 days in group T and 6.93 ± 2.71 days in group P. The difference was not statistically significant (*P* = n.s.). There were no instances of DVT or any other adverse reactions for blood transfusion in study patients.

## 4. Discussion

Our findings reject the null hypothesis of the study and showed that usage of low dose perioperative TA reduces blood loss and the need for blood transfusion in patients undergoing primary unilateral TKA. In this study we evaluated the effect of the reception of 500 mg intravenous TA once before and once after surgery on postoperative blood loss by means of measuring the Hb levels which was done several times after surgery. Another method was measuring the drain output in the first 48 hours after surgery. It was found that twice receiving 500 mg of TA, once 30 minutes before surgery, and once three hours after surgery significantly reduces the loss of Hb, drain volume, and need for blood transfusion. However, it does not reduce the hospitalization period of patients.

There are some previous studies that have evaluated the impact of intravenous TA on blood loss after TKA with different doses and regimens. These studies can be divided into three groups. In the first group there are studies that have evaluated the effectiveness of low dose regimens of TA. The administration dose in this group of studies was 10–15 mg/Kg (1 gram or lower). Hiippala et al. administered 15 mg/Kg of TA and found a significant decrease in drain volume and transfusion rate but did not find a significant decrease in the drop of Hb levels [13].

Sarzaeem et al. conducted a well-designed double-blind randomized clinical trial which showed that administration of a single 500 mg dose of TA significantly reduces the need for blood transfusion and reduces Hb drop and drain output after surgery [14]. In another randomized trial, Maniar et al. compared four methods of TA administration and showed that a single dose of intravenous TA did not give effective results [15]. In other studies Orpen et al. [16] and McConnell et al. [17] did not find significant reduction in Hb drop, transfusion rate, and drain output while Lin et al. [18] administered 10 mg/Kg of TA and showed significant reduction in total blood loss and transfusion rate. As can be seen, there is no consensus among different studies. However, it is the authors' belief that the reason in some studies no significant changes were seen in the outcome was because of their low sample sizes and that studies with larger sample sizes showed the effectiveness of TA.

In the second group there are studies that evaluated the intermediate dose regimens (1-2 grams). Most of these studies administered 10–15 mg/kg for the first dose and another 10–15 mg/kg during the first 12 hours after surgery [10, 19–27].

In the last group there are studies in which high doses of TA (more than 2 grams) were administered [28–30]. They administered TA with different protocols in doses larger than those of the second group. Jansen et al. used 15 mg/kg of TA before the surgery and the same dose every 8 hours for 3 days after surgery and found a significant reduction in Hb level drop [30]. In another study Dahuja et al. [28] used the same regimen for 2 days and reached results similar to those achieved by Jansen el al. Most studies in the second and third group revealed that TA is effective in reducing blood loss and Hb drop after surgery, a result studies in the first group did not reach.

Another important variable that was evaluated in this study was the hospitalization period of study patients. Few of the previous studies have considered this item and most of them concluded that TA may reduce the duration of hospital stay [31, 32]. In this study it was found that although TA reduces Hb drop and the need for transfusion, it does not have a significant impact on the period of hospitalization. This issue should be considered in further cost effectiveness studies for the routine use of TA in TKA.

One of the strengths of this study is that all surgeries were performed by a single expert surgeon and a single prosthetic brand was used which excludes the possible role of technical effects on our results.

This study also had some downsides. First of all, intraoperative bleeding was not evaluated. Other downsides are that the diagnosis of TA complications such as DVT or PTE was based on clinical signs and symptoms, not the gold standard diagnostic tests, and that other nonthrombotic adverse events of TA were not evaluated.

In conclusion it is deduced that perioperative intravenous administration of TA in two 500 mg doses, first dose before and second dose three hours after surgery, reduces Hb drop and drain output volume and the need for blood transfusion after TKA. The failure of previous studies to show this effect is due to their low sample size and study design. On the other hand this study showed that low dose perioperative intravenous TA does not influence the hospitalization period.

## Figures and Tables

**Table 1:** Demographic variables between study groups.

Variable	Group T (*n* = 45)	Group P (*n* = 45)	*P* value
Age (year)	67.04 ± 8.01	65.66 ± 4.97	n.s.
Sex			
Women	35 (77.78%)	32 (71.12%)	n.s.
Men	10 (22.22%)	13 (28.88%)	

Data are presented as mean ± standard deviation and number (%). n.s.: nonsignificant. *P* < 0.05 is considered statistically significant.

**Table 2:** Hb level before and during the study period between study groups.

Hb level (g/dL)	Group T	Group P	*P* value
Before surgery	12.90 ± 1.11	12.63 ± 1.24	n.s.
6 hours after surgery	11.77 ± 1.26	11.21 ± 1.31	0.04^*∗*^
24 hours after surgery	11.01 ± 1.17	9.96 ± 1.15	0.001^*∗*^
48 hours after surgery	10.92 ± 0.97	10.23 ± 0.98	0.001^*∗*^

Data are presented as mean ± standard deviation. n.s.: nonsignificant. ^*∗*^ *P* < 0.05 is considered statistically significant.
